# The reduction of post-cardiac surgery infections by statins: solid evidence?

**DOI:** 10.1007/s12471-014-0605-1

**Published:** 2014-10-02

**Authors:** S. C. A. M. Bekkers

**Affiliations:** Department of Cardiology, Maastricht University Medical Center, P. Debyelaan, 25 PO Box 5800, 6202 AZ Maastricht, the Netherlands

Infectious complications following cardiac surgery are associated with increased mortality, prolonged hospital stay and increased health care costs [1]. The prevalence of post-cardiac surgery infections ranges between 5 and 21 %, depending on whether surgical site (sternal wound and leg harvest site infections) or non-surgical site infections (pneumonia, urinary tract infection, bacteraemia, Clostridium colitis) are taken into account [2]. The incidence of post-cardiac surgery infections is expected to increase with an ageing population and the increased use of cardiac surgery in higher risk groups, such as diabetics, obese patients, and patients undergoing repeat surgery. Consequently, early identification of high-risk patients and implementing risk-reducing interventions are becoming increasingly important.

Statins are frequently prescribed drugs with widely accep-ted cardiovascular benefits. Beyond cholesterol lowering, they are also recognised for their anti-inflammatory and immune-modulating properties. Because of these so-called pleiotropic effects, statins may be beneficial for the prevention of post-cardiac surgery infectious complications. In this issue of the journal, Hartholt et al. report in a retrospective cohort analysis of 520 patients undergoing cardiothoracic surgery (coronary artery bypass grafting [CABG], valve surgery, aortic surgery, or other), that preoperative statin therapy was associated with a 67 % reduced risk of postoperative infections (adjusted odds ratio 0.33. 95 % CI 0.19–0.57, *p* < 0.001) [3]. Overall, postoperative infections occurred in 15.8 % of patients (12.2 % in statin group and 24.7 % in control group, *p* = 0.001). The association of preoperative statin therapy with reduced postoperative infections exclusively concerned non-surgical site but not surgical site infections. In addition, reduced in-hospital mortality was found in preoperative statin users (3.2 % versus 7.3 %, *P* = 0.041).

At first glance, these results appear promising but they should be placed in a broader perspective. Results of previous observational studies and meta-analyses evaluating the association of preoperative statin therapy and post-cardiac surgery infections have been mixed and conflicting, as the authors acknowledge [4–7]. Compared with previous ‘positive association’ studies, the current study by Hartholt et al. shows remarkable and difficult to explain differences. Although a small cohort study compared with others (520 versus 1934 and 6253 patients), postoperative infections occurred twice as often (15.8 % versus 7.2 % and 7.8 %), while the observed risk reduction was largest (67 % versus 26 % and 33 %) [6, 7]. In the study by Kayani et al., preoperative statin therapy was associated with an overall reduction of post-cardiac surgery infections, and mainly concerned a reduction of surgical site infections but not pneumonia and sepsis. This is in contrast with the study by *Hartholt* et al., who report a reduction in pneumonia and urinary tract infections instead. These divergent results can be explained by methodological deficiencies of observational studies in general. Observational studies demonstrate associations rather than causal relationships and despite the availability of accurate statistical adjustment there is always the risk of unmeasured confounding. Some of these confounding variables include 1) the prescription of statins to patients with high cholesterol, which is in itself associated with a lower risk of infection, and 2) the positive selection of ‘healthier’ patients because of their better tolerance to statins (less healthy patients will be less likely to take statins). Randomised placebo-controlled clinical trials investigating the true effect of preoperative statin therapy and post-cardiac surgery infections are currently lacking. A recent meta-analysis investigated data from 11 randomised placebo-controlled statin trials (30,947 patients, 45.6 % receiving statins) that were designed to mainly investigate cardiovascular events, but reported infectious complications as well. Interestingly, the meta-analysis did not find evidence for a reduced risk of infections and infection-related mortality in patients taking statins (relative risk 1.00 (95 % CI 0.96–1.05) and 0.97 (95 % CI 0.83–1.13), respectively) [8].

The mechanism by which statins could exert their infection-reducing effects remains speculative. Although statins have anti-inflammatory and immune-modulating effects, they have no direct antibacterial effects and are by no means antibiotics. In vitro studies of the anti-inflammatory effects of statins are misleading, because the minimum inhibitory concentrations needed far exceed pharmacological levels that can be achieved with usual human doses [9]. Furthermore, if statins really reduce the risk of postoperative infections, why do they exert this protective effect mainly when prescribed preoperatively? Indeed, Hartholt et al. showed that postoperative initiation of statin therapy (in 7.1 % of patients) was not associated with a reduced risk of infection.

For sure, Hartholt et al. are complimented on their positive findings but their study adds to a list of existing observational studies and does not give us the definitive answer whether statins truly reduce postoperative infections. Although large randomised clinical trials could help to solve this problem, it is questionable whether these are doable. Because many patients undergoing cardiac surgery (especially CABG) have a class I indication for statins and stopping statins would be unethical, the study would have to be limited to patients without coronary artery disease undergoing valve surgery only [10]. Furthermore, designing such a randomised clinical trial is complicated because the type, dose and time point when to start statin before surgery are currently unknown. For now, there is still insufficient evidence that statins reduce infections.
